# Supplementary material for: Nicotinamide Attenuates the Progression of Renal Failure in a Mouse Model of Adenine-Induced Chronic Kidney Disease
Source: Toxins (Basel). 2021 Jan 11;13(1):50. doi: 10.3390/toxins13010050 (PMC7827863; doi:10.3390/toxins13010050)
Supplement: Supplementary file 1 [file toxins-13-00050-s001.pdf]

Nicotinamide Attenuates the Progression of Renal Failure in a Mouse Model of Adenine-induced Chronic Kidney Disease

Satoshi Kumakura, Emiko Sato, Akiyo Sekimoto, Yamato Hashizume, Shu Yamakage, Mariko Miyazaki, Sadayoshi Ito, Hideo Harigae and Nobuyuki Takahashi

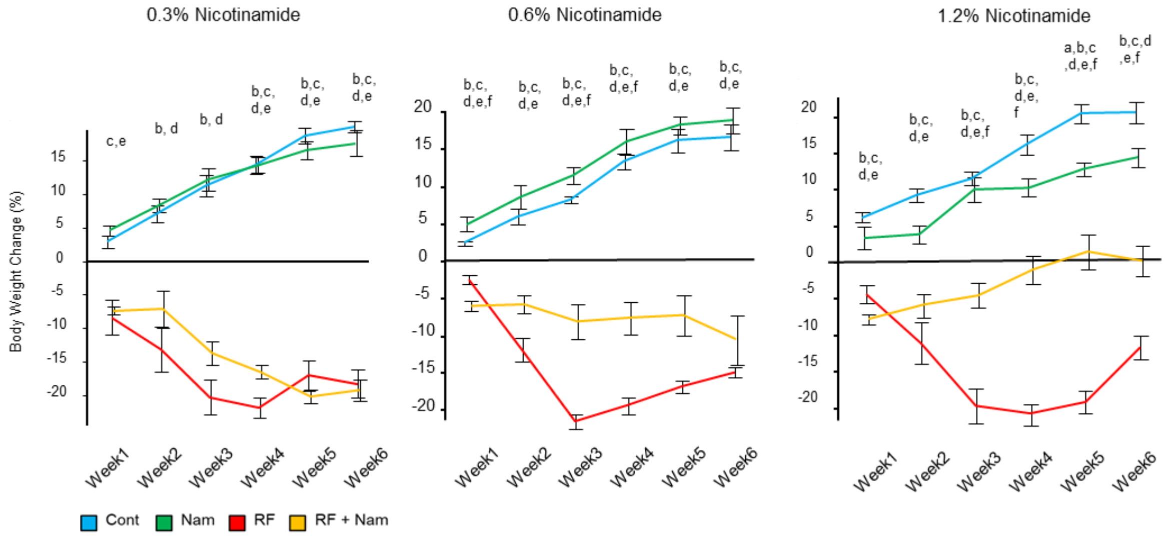

Figure S1. The percentage of body weight referred to baseline in the nicotinamide prophylactic administration. Values are means ± SEM; Steel-Dwass test: a: Cont vs. Nam, b: Cont vs. RF, c: Cont vs. RF + Nam, d: Nam vs. RF, e: Nam vs. RF + Nam, f: RF vs. RF + Nam, $p < 0.05$. Control diet without Nam (Cont), control diet plus Nam (Nam), adenine-rich diet without Nam (RF) and adenine-rich diet plus Nam (RF + Nam).

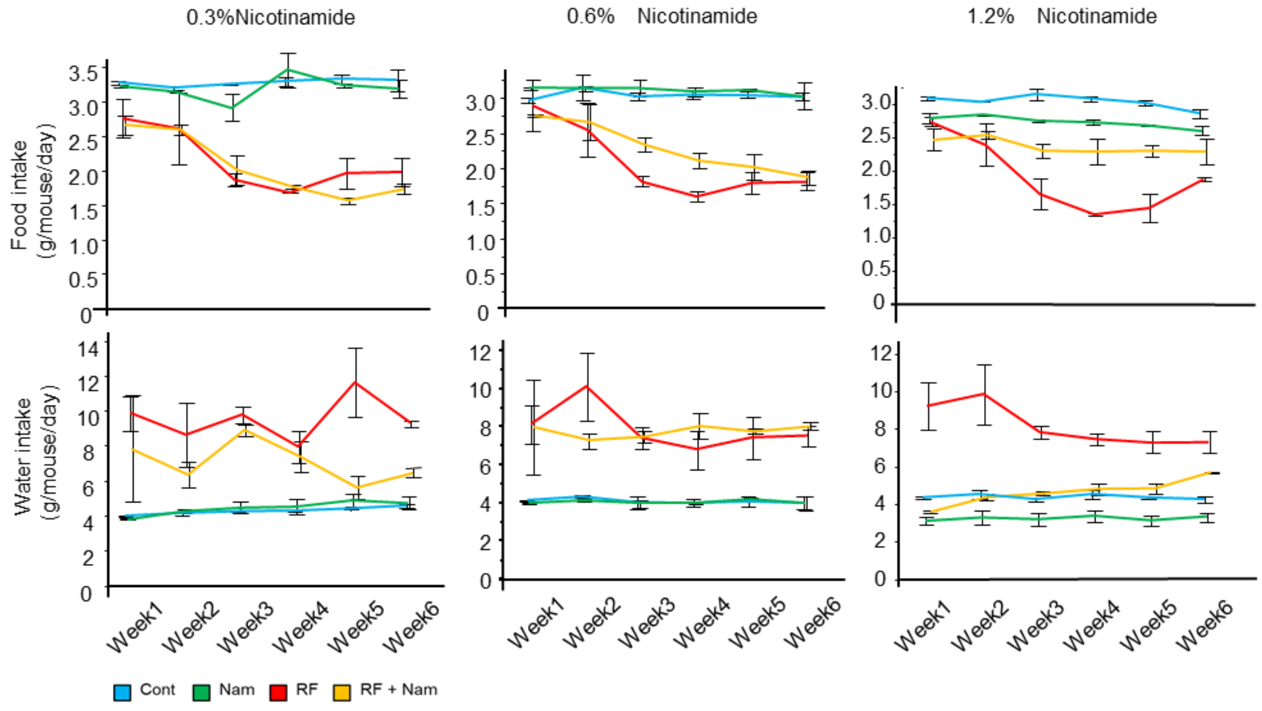

Figure S2. The weekly average of food and water in the nicotinamide prophylactic administration. Values are means ± SEM. Control diet without Nam (Cont), control diet plus Nam (Nam), adenine-rich diet without Nam (RF) and adenine-rich diet plus Nam (RF + Nam).

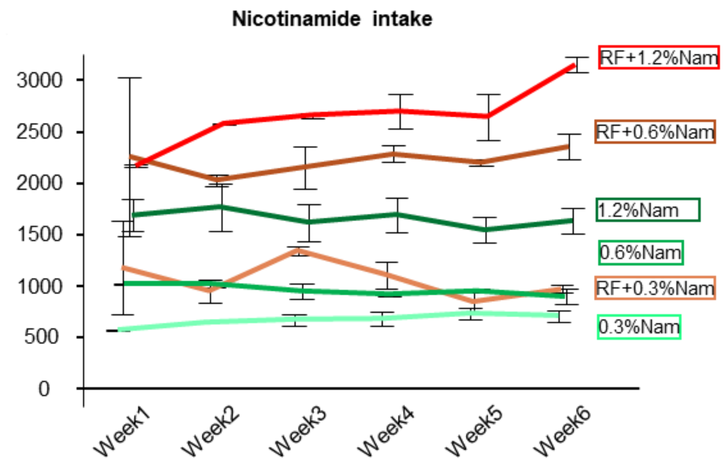

Figure S3. The average of daily nicotinamide intake for each experimental week in the nicotinamide prophylactic administration experiment. Control diet plus Nam (Nam) and adenine-rich diet plus Nam (RF + Nam).

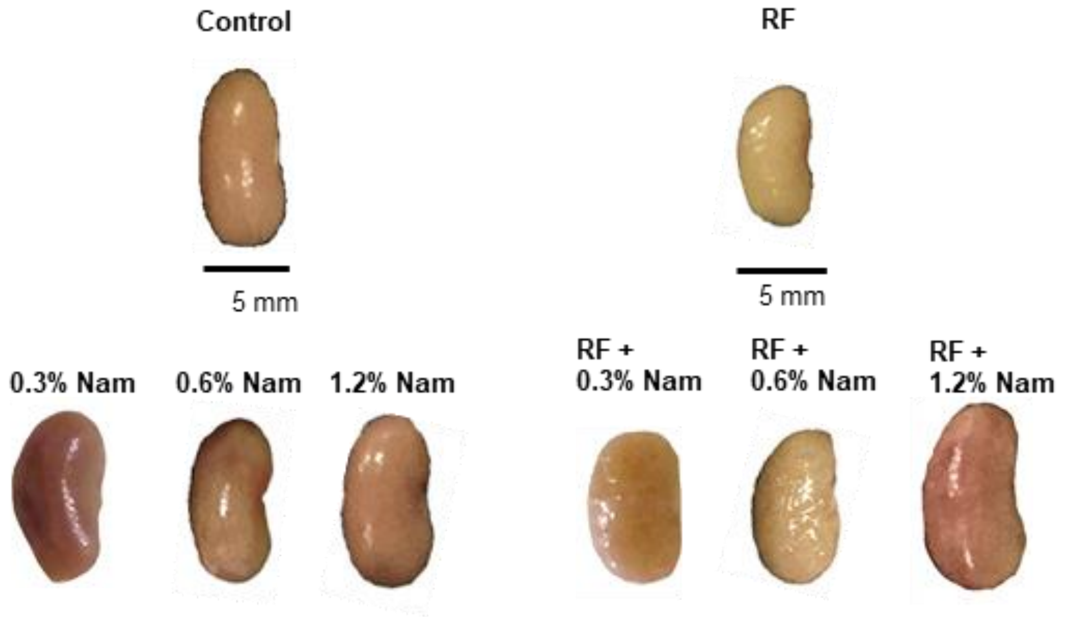

Figure S4. The representative image of harvested left kidneys at 6th week. Kidneys were harvested immediately after saline perfusion. Control diet without Nam (Cont), control diet plus Nam (Nam), adenine-rich diet without Nam (RF) and adenine-rich diet plus Nam (RF + Nam).

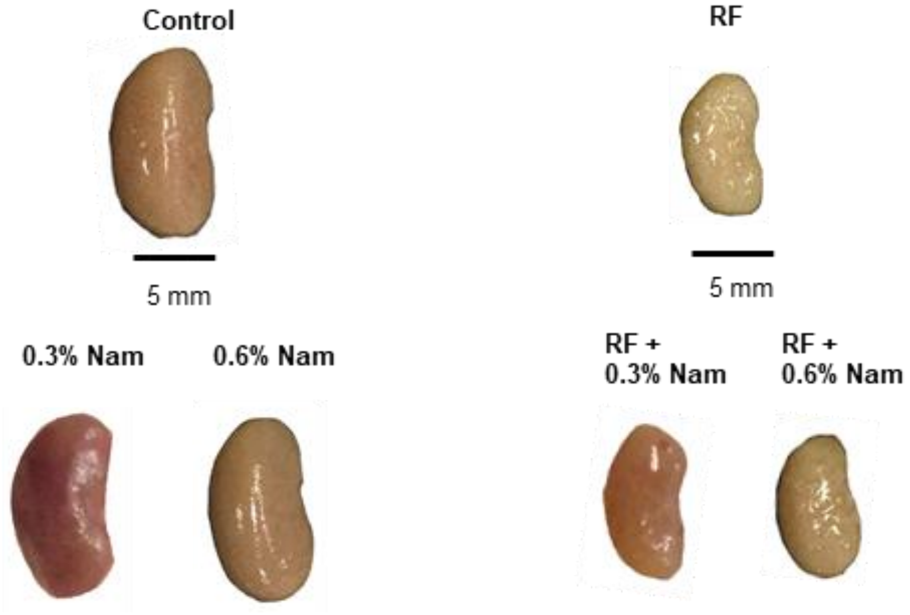

Figure S5. The representative image of harvested left kidneys at 10th week. Kidneys were harvested immediately after saline perfusion. Control diet without Nam (Cont), control diet plus Nam (Nam), adenine-rich diet without Nam (RF) and adenine-rich diet plus Nam (RF + Nam).

Table S1. Body weight & Organ weight at 6th week of Prophylactic nicotinamide administration.

0.3% Nicotinamide

	**Cont**			**Nam**			**RF**			**RF + Nam**			**ANOVA**
	**mean**		**SD**	**mean**		**SD**	**mean**		**SD**	**mean**		**SD**	***p*-value**
Body Weight (g)	27.8	±	1.3	27.3	±	2.4	19.1	±	1.6	18.5	±	1.1	<0.0001
Brain (mg)	464.2	±	3.7	455.7	±	12.5	427.0	±	8.5	410.4	±	7.8	<0.0001
Thymus (mg)	46.7	±	10.7	37.7	±	4.3	37.6	±	54.7	12.0	±	5.4	<0.0001
Heart (mg)	13.8	±	11.2	140.0	±	18.1	107.9	±	7.4	95.9	±	9.0	<0.0001
Lung (mg)	152.0	±	31.5	171.3	±	60.1	177.9	±	59.8	137.3	±	47.4	0.5
Right Kidney (mg)	169.3	±	10.5	173.5	±	14.6	103.0	±	22.0	120.6	±	15.5	<0.0001
Left Kidney (mg)	161.7	±	22.5	159.3	±	20.7	103.4	±	16.1	123.7	±	11.8	<0.0001
Adrenal gland (mg)	7.7	±	1.2	8.8	±	2.1	7.1	±	1.6	5.7	±	1.3	0.01
Cecum (mg)	159.8	±	9.5	162.8	±	35.0	150.6	±	24.7	135.9	±	21.5	0.2
Colon (mg)	168.5	±	26.8	147.5	±	20.9	112.3	±	23.9	94.9	±	17.5	<0.0001
Liver (mg)	1412.2	±	327.9	1214.5	±	154.5	738.9	±	307.1	800.9	±	34.7	<0.0001
Pancreas (mg)	281.3	±	31.6	280.3	±	22.4	264.1	±	52.4	195.6	±	15.6	0.0003
Spleen (mg)	69.8	±	8.9	68.8	±	8.2	71.6	±	7.8	60.0	±	10.0	0.1
WAT (mg)	696.2	±	202.5	537.2	±	78.8	100.1	±	53.7	76.7	±	33.3	<0.0001
BAT (mg)	85.3	±	17.0	89.0	±	10.8	54.3	±	4.5	45.0	±	3.7	<0.0001

0.6% Nicotinamide

	**Cont**			**Nam**			**RF**			**RF + Nam**			**ANOVA**
	**mean**		**SD**	**mean**		**SD**	**mean**		**SD**	**mean**	**±**	**SD**	***p*-value**
Body Weight (g)	25.6	±	1.3	26.5	±	0.8	18.6	±	1.5	20.4	±	1.6	<0.0001
Brain (mg)	448.9	±	17.8	438.9	±	13.2	423.0	±	8.4	423.2	±	12.8	0.004
Thymus (mg)	35.4	±	5.1	41.2	±	6.5	10.4	±	7.3	21.4	±	8.7	<0.0001
Heart (mg)	134.5	±	12.5	131.3	±	6.3	101.7	±	9.4	104.9	±	8.5	<0.0001
Lung (mg)	154.2	±	36.1	205.5	±	79.5	201.0	±	76.8	162.7	±	63.8	0.4
Right Kidney (mg)	155.4	±	6.7	156.7	±	5.4	106.2	±	27.5	124.2	±	7.9	<0.0001
Left Kidney (mg)	155.3	±	20.5	149.6	±	11.6	96.8	±	11.0	133.8	±	11.5	<0.0001
Adrenal gland (mg)	5.4	±	0.8	6.3	±	1.9	7.0	±	1.2	5.9	±	0.7	0.1
Cecum (mg)	128.6	±	27.5	123.2	±	20.1	120.3	±	27.1	138.1	±	27.6	0.6
Colon (mg)	156.8	±	10.0	144.0	±	14.8	111.0	±	10.2	101.5	±	23.3	<0.0001
Liver (mg)	1074.0	±	75.3	1143.0	±	24.6	829.2	±	104.1	909.9	±	156.2	<0.0001
Pancreas (mg)	212.0	±	62.5	214.6	±	29.5	218.7	±	35.8	166.6	±	38.7	0.1
Spleen (mg)	56.7	±	4.4	58.5	±	5.2	56.0	±	9.1	69.0	±	9.1	0.02
WAT (mg)	605.9	±	167.8	572.8	±	92.2	158.9	±	195.3	164.7	±	82.6	<0.0001
BAT (mg)	79.9	±	26.7	81.3	±	16.2	51.2	±	17.8	53.0	±	11.9	0.006

1.2%Nicotinamide

	**Cont**			**Nam**			**RF**			**RF + Nam**			**ANOVA**
	**mean**		**SD**	**mean**		**SD**	**mean**		**SD**	**mean**		**SD**	***p*-value**
Body Weight (g)	25.5	±	1.0	24.4	±	1.4	18.8	±	1.1	21.7	±	1.7	<0.0001
Brain (mg)	437.3	±	51.6	448.7	±	19.5	418.3	±	12.0	431.0	±	11.1	0.3
Thymus (mg)	53.4	±	14.2	53.7	±	12.8	27.6	±	11.1	45.2	±	16.7	0.004
Heart (mg)	134.4	±	13.1	135.5	±	13.6	100.3	±	5.5	117.5	±	8.2	<0.0001
Lung (mg)	165.2	±	24.3	187.5	±	43.0	194.6	±	65.5	206.0	±	59.4	0.6
Right Kidney (mg)	164.9	±	10.0	158.9	±	20.1	94.5	±	12.1	139.7	±	25.2	<0.0001
Left Kidney (mg)	151.9	±	16.6	151.2	±	18.4	94.3	±	15.6	144.5	±	15.3	<0.0001
Adrenal gland (mg)	6.0	±	3.1	4.3	±	1.5	5.2	±	1.5	5.8	±	1.6	0.5
Cecum (mg)	135.3	±	17.1	176.1	±	63.9	109.0	±	29.8	124.3	±	19.6	0.02
Colon (mg)	160.1	±	26.8	137.0	±	26.8	137.1	±	33.0	137.9	±	10.2	0.4
Liver (mg)	1183.7	±	120.5	1293.6	±	224.3	913.6	±	96.7	975.8	±	79.2	0.0001
Pancreas (mg)	192.0	±	12.3	166.7	±	17.9	170.9	±	43.4	161.7	±	31.6	0.4
Spleen (mg)	63.4	±	5.8	52.2	±	0.7	61.5	±	10.3	63.7	±	10.2	0.5
WAT (mg)	455.6	±	86.1	419.7	±	46.0	97.6	±	22.7	248.5	±	46.9	<0.0001
BAT (mg)	80.5	±	18.3	59.1	±	15.7	44.7	±	10.1	57.8	±	20.8	0.005

Abbreviations: WAT white adipose tissue, BAT brown adipose tissue.

Table S2. Body weight & Organ weight at 10th week of nicotinamide administration in advanced disease.

0.3% Nicotinamide

	Cont			Nam			RF			RF + Nam			ANOVA
	**mean**		**SD**	**mean**		**SD**	**mean**		**SD**	**mean**		**SD**	***p*-value**
Body Weight (g)	29.3	±	2.2	29.0	±	1.3	25.6	±	1.6	22.6	±	1.4	<0.0001
Brain (mg)	452.0	±	24.4	461.4	±	21.8	441.3	±	20.5	424.8	±	24.6	0.03
Thymus (mg)	39.1	±	7.0	37.7	±	6.7	44.9	±	5.7	31.3	±	8.5	0.01
Heart (mg)	147.6	±	24.8	142.9	±	6.8	144.3	±	14.4	120.8	±	13.1	0.01
Lung (mg)	159.7	±	20.7	210.4	±	84.0	199.4	±	103.1	166.5	±	63.5	0.5
Right Kidney (mg)	179.0	±	10.8	178.9	±	9.7	88.4	±	7.7	90.9	±	9.0	<0.0001
Left Kidney (mg)	164.1	±	7.2	170.9	±	7.2	94.6	±	18.7	93.1	±	5.4	<0.0001
Adrenal gland (mg)	8.7	±	2.1	5.6	±	1.5	8.3	±	1.8	7.8	±	1.0	0.007
Cecum (mg)	151.7	±	23.0	141.7	±	21.5	155.7	±	21.5	156.8	±	18.9	0.5
Colon (mg)	196.7	±	44.7	166.4	±	26.8	191.7	±	14.7	139.6	±	21.7	0.003
Liver (mg)	1329.1	±	80.6	1323.9	±	116.0	1260.1	±	125.3	1095.1	±	124.4	0.001
Pancreas (mg)	299.0	±	32.9	293.7	±	33.5	301.1	±	44.6	253.4	±	21.2	0.03
Spleen (mg)	79.3	±	6.9	70.7	±	5.0	102.0	±	7.7	74.8	±	10.6	<0.0001
WAT (mg)	820.4	±	293.7	865.1	±	195.6	301.3	±	60.6	246.6	±	76.4	<0.0001
BAT (mg)	130.1	±	38.4	99.6	±	10.7	102.0	±	25.2	67.3	±	7.4	0.0003

0.6% Nicotinamide

	Cont			Nam			RF			RF + Nam			ANOVA
	**mean**		**SD**	**mean**		**SD**	**mean**		**SD**	**mean**		**SD**	***p*-value**
Body Weight (g)	27.0	±	1.0	27.7	±	1.6	24.9	±	0.6	17.6	±	1.0	<0.0001
Brain (mg)	453.9	±	9.0	460.6	±	12.1	448.6	±	10.6	409.4	±	11.3	<0.0001
Thymus (mg)	36.1	±	5.8	63.5	±	88.2	47.3	±	5.8	27.2	±	47.3	<0.0001
Heart (mg)	144.0	±	13.9	148.8	±	11.2	146.3	±	8.5	92.3	±	8.3	<0.0001
Lung (mg)	156.9	±	49.9	157.5	±	31.7	136.4	±	11.3	120.8	±	24.4	0.1
Right Kidney (mg)	169.0	±	6.7	170.2	±	19.3	90.0	±	9.3	83.5	±	11.3	<0.0001
Left Kidney (mg)	154.3	±	12.8	160.8	±	11.2	89.8	±	13.2	88.2	±	11.8	<0.0001
Adrenal gland (mg)	6.5	±	1.1	6.9	±	2.4	7.6	±	1.6	6.6	±	1.2	0.6
Cecum (mg)	171.8	±	34.7	170.5	±	38.0	177.8	±	40.1	131.1	±	17.7	0.06
Colon (mg)	166.0	±	21.9	135.4	±	26.4	144.6	±	32.9	91.6	±	16.8	0.0002
Liver (mg)	1245.5	±	69.6	1248.2	±	87.7	1108.6	±	73.7	870.0	±	75.6	<0.0001
Pancreas (mg)	290.9	±	20.9	268.6	±	37.2	296.8	±	19.5	173.6	±	30.8	<0.0001
Spleen (mg)	65.0	±	4.3	67.3	±	5.9	78.4	±	6.0	39.0	±	5.5	<0.0001
WAT (mg)	610.8	±	44.2	609.8	±	228.9	300.9	±	36.1	107.8	±	44.0	<0.0001
BAT (mg)	110.9	±	25.8	98.2	±	12.9	98.4	±	8.8	72.1	±	28.1	0.02

Abbreviations: WAT white adipose tissue, BAT brown adipose tissue.

Table S3. Concise method and settings of GC-MS measurements.

Analyte	Retention time (min)	Ion (MS)
Glucose-meto-5TMS	13.391	319.1, 205.1
Glucose 6-phospahte-meto-6TMS	16.002	471.2, 387.2
Ribulose 5-phosphate-meto-5TMS	14.724	357.1, 299.1
Ribose 5-phosphate-meto-5TMS	14.749	459.2, 315.1
Fructose 6-phosphate-meto-6TMS	15.915	459.2, 357.1
3-Phosphoglycerate-4TMS	12.826	459.1, 387.2
2-Phosphoglycerate-4TMS	12.62	459.1, 387.2
Phosphoenolpyruvate-3TMS	11.324	369.1, 299.1
6-Phosphogluconate-7TMS	16.565	387.1, 357.1
Pyruvate-oxime-2TMS	7.175	232.0, 247.0
Lactic acid-2TMS	6.173	219.0, 191.0
Citric acid-4TMS	12.921	363.0, 347.0
Isocitric acid-4TMS	12.914	465.0, 375.0
2-Ketoglutaric acid-meto-2TMS	11.177	198.1, 288.1
Succinyl acid-2TMS	8.901	247.0, 172.0
Fumaric acid-2TMS	9.244	245.0, 217.0
Malic acid-3TMS	10.392	233.0, 189.0
Citric acid-d_4-4TMS	12.899	367.2, 350.1
2-Isopropylmalic acid-3TMS	11.128	349.1, 377.1
